# Prevalence of adolescent-reported food insecurity and the determinants including coping strategies living in urban slum communities of Bangladesh during the era of COVID-19: a cross-sectional study

**DOI:** 10.1186/s12889-023-16984-y

**Published:** 2023-10-19

**Authors:** SM Taniya Yasmin, SM Taslima Yasmin, Sarah Sultan, Seo Ah Hong

**Affiliations:** 1 ASEAN Institute for Health Development, Mahidol University, Nakhon Pathom, 73170 Thailand; 2 Social and Economic Enhancement Program, Mirpur, Pallabi, Dhaka 1216 Bangladesh

**Keywords:** Adolescents, Food insecurity, Coping strategies, Bangladesh, COVID-19 pandemic

## Abstract

**Background:**

As food insecurity (FI) continues to rise worldwide especially in developing countries like Bangladesh, adolescent experience of FI have received minimal attention globally. This study aimed to identify the prevalence of adolescent-reported FI and its association with individual and socio-environmental factors as well as coping strategies amongst a sample of adolescents living in urban slum areas of Bangladesh in the times of the Coronavirus 19 (COVID-19) pandemic.

**Methods:**

A descriptive cross sectional study was conducted amongst 326 adolescents (12–18 years) living in the urban slums of Narayanganj, Dhaka from April to May, 2022. Adolescent-reported FI was assessed using a structured questionnaire adopted from Household Food Insecurity Access Scale (HFIAS). Descriptive statistics, Chi-square tests and ordinal logistic regression were used to draw inference.

**Results:**

Prevalence of adolescent-reported FI was high (46.6% moderate and 29.8% severe). The likelihood of experiencing moderate or severe FI versus no/mild FI were 1.7 times (95% Confidence Interval (CI) [1.1, 2.5]) higher in younger adolescents and 5 times (95% CI [2.3, 12.7]) higher in unemployed youth. Socio-environmental factors determining the economic status of a household such as higher number of family members, only one earning family member, unemployed father, no household assets, food aid received by the family during pandemic and positive COVID-19 infection in family were associated with moderate and severe FI. Coping strategies such as a higher number of food seeking strategies (Adjusted Odds Ratio (AOR) 3.4, 95% CI [1.9, 5.9]), substance use (AOR 6.2, 95% CI [1.2, 31.7]) and stopping school (AOR 3.3, 95% CI [1.9, 5.7]) increased odds for moderate and severe FI. Stratified by drop-out of school status, an association between food seeking strategies and FI remained significant among those school-going, while there was no association among those dropping out of school.

**Conclusion:**

This study showed that adolescents living in urban slum communities in Bangladesh are at very high risk of FI and resort to harmful coping strategies. Our study highlights the importance of further research in adolescent reported FI and coping strategies in low to middle income countries (LMICs) and create appropriate interventions to lower FI among this group and improve their state of health and wellbeing.

## Background

Food insecurity (FI) is defined by Food and Agricultural Organization of United Nations as “a situation that exists when people lack regular access to adequate amount of safe and nutritious food for normal growth and development and an active and healthy life” [1]. During the past decade, there was a steady global rise in the level of moderate to severe food insecure people, which accounts for almost 30% of the world’s population, with more than half of them hailing from Asia and one third from Africa [1]. In addition, the COVID-19 pandemic, along with its lingering impact on global economies and the unrecovered income losses among those most affected by the pandemic, particularly in low to middle income countries (LMICs) like Bangladesh, has exacerbated the state of food insecurity and hindered progress towards meeting Sustainable Development Goal (SDG) 2 by 2030 [1]. Many studies on FI in developed [2] and developing countries [3, 4] showed that FI is associated with poor health outcomes among all age groups but emphasis has largely been on the vulnerability of households and young children [5–8]. Furthermore, since studies reported that parents often protected younger children than older children from the impacts of household FI [9], adolescents often face a higher vulnerability to FI. Pre-pandemic, Around 50% of adolescents in developing countries were already suffering from FI [10, 11].

Since adolescence is a crucial transitional period between childhood and adulthood, it is characterized by major physical, psychological, and social transitions. Thus, FI during this period results in not only food related health outcomes, such as micronutrients deficiency and poor health outcomes [5, 12], but also consist of anxiety, feeling of distress and deprivation, as well as adverse changes in family and social interactions [2, 13]. Nonetheless, the adolescent FI has received less attention particularly in LMICs such as Bangladesh. Very little is known about adolescent FI especially among adolescents hailing from disadvantaged backgrounds, such as urban slum settings, although they are disproportionately affected by a higher burden of health risks since childhood [12, 14–16].

When individuals are food insecure, they resort to coping strategies which minimize risks to an individual’s food and economic resources in times of crisis [17]. Thus, the type of coping strategies used may indicate a severity of FI [18]. Previous research showed that adolescents used some strategies to cope with FI, such as selling drugs, asking for food, stealing and borrowing money/food [2, 19], and they may be associated with higher chances of dropping out from school [2] and engaging in unhealthy behaviors, such as smoking and using drugs [20], ultimately contributing to social disadvantage when transitioning to adulthood [21]. Literature on adolescent-specific health outcomes provide a strong rationale for research on FI and coping strategies. In particular, a better understanding of the implemented coping strategies by adolescents suffering from FI in extremely food-insecure environments like the urban slums may be beneficial in developing appropriate and effective programs.

Since most studies on adolescent FI were assessed based on parent’s reports, the estimate based on parental accounts often failed to adequately gauge the adolescents’ experiences and underestimated FI in adolescents [21, 22]. With a dearth of adequate information regarding self-reported FI experience and coping strategies among either school-going or working adolescents in LMICs like Bangladesh, this study aimed to identify the prevalence and determinants of FI and their association with implemented coping strategies among adolescents living in urban slums of Narayanganj, Dhaka, Bangladesh and also to identify the association between coping strategies and FI amongst school going and dropout adolescents during the era of COVID-19 pandemic. Bangladesh has an urban population of nearly 65 million people and about half of them are slum dwellers [23]. Dhaka Division has been recorded to have the highest number of slum settlements and in terms of population density, Narayanganj has the greatest proportion of slum households [24]. Prioritizing the needs of underprivileged adolescents, allows us to lay the foundation for a healthier, prosperous and more equitable future. This study can serve as a premise for further research and discussion on adolescent FI in urban slum communities in Bangladesh and other LMICs because there is minimal awareness regarding this public health crisis.

## Methods

### Study design and participants

The study, a quantitative cross-sectional study, took place from April to May 2022 in urban slums of Narayanganj district of Dhaka division via face to face interview using structured questionnaire in Bengali language. We purposively chose Narayanganj, as it has one of the highest rates of urban population growth in Dhaka division with most of them residing in urban slums [25]. Narayanganj was the epicenter of the COVID-19 in Bangladesh and faced one of the strictest lockdown measures during the pandemic, which severely affected the already struggling slum dwellers [26]. Adolescents from 12 to 18 years of age, either working or school going, were recruited for this study. Based on Cochran’s formula, the sample size was estimated using 95% confidence interval, a precision of 0.05, and a prevalence of FI as 25% [27], and anticipating 10% response/missing data, the determined sample size was 316. A total of 326 adolescents were recruited for this study.

Of 27 wards in Narayanganj City Corporation, three wards were selected via random lottery sampling and two slums per ward were selected via convenience sampling. Lists of adolescents and their households from the selected slums were obtained by the researchers from government and non-governmental organizations who were working with households in the slum areas and numbered. Equal number of adolescents per slum were then selected via random sampling for the survey. Selected adolescents and their households were then approached for consent/assent to participate in a face to face survey. If researchers failed to contact/approach selected participant, another participant was selected randomly to participate for the survey.

Prior to undertaking the study, ethical approval was obtained from a Mahidol University Ethical Committee (2022/03–054) and Ward Councilors. Pretesting the questionnaire was performed on around 5% of the total sample size at none of the selected slums and minimal corrections were required. COVID-19 precautions were maintained during the survey by both researchers and participants by wearing masks and maintaining social distance. After explanation of study objectives and process, written informed assent was obtained from the adolescents along with written consent from the guardian with assurance of confidentiality. All data were treated anonymously using study identification numbers.

### Measures

The FI was assessed using the Household Food Insecurity Access Scale (HFIAS), which has been validated for use in several LMICs [28, 29]. To measure adolescent FI, we adopted the HFIAS questions from a previous study in Pakistan [10]. The Chronbach’s alpha coefficient was used to assess internal consistency (reliability) of the FI question items and the coefficient was 0.82. Four ordinal categories of FI were developed depending on the obtained score: 0–1 (no FI), 2–8 (mild FI), 9–16 (moderate FI) and 17–27 (severe FI) [28].

As current literatures lack standardized questionnaires to identify coping strategies for FI in adolescents, a structured coping strategy questionnaire was designed by the researchers based on literature review with three coping strategies: (1) food seeking strategies, (2) dropping out of school due to lack of food/money, and (3) substance use. During the past 30 days, when you did not have enough food or money to buy food, how often the following seven food-seeking strategies adolescents used were asked: (i) acquiring food on loan/credit, (ii) visiting neighbors/relatives for food, (iii) storing food for self, (iv) searching for food outside home (v) borrowing money from others to acquire food, (vi) shoplifting, and (vii) begging for foods/money [2, 19, 21, 30]. The responses (‘never’=0, ‘sometimes’=1, and ‘often/always’=2) were scored. A total score was then obtained for each participant by adding up the scores of individual strategies (0 to 14 scores) and categorized into three equal groups using tertiles. Second, whether the adolescent has dropped out of school due to lack of food/money to save expenses were asked and the response was recorded as “yes” or “no”. Lastly, during the past 30 days, three questions about ‘how many days did you use the following substance’ were asked: (i) smoking, (ii) any other smokeless tobacco products (e.g. biri, jarda, tobacco leaf, gul, or shisha), and (iii) marijuana (also called ganja or weed), adopted from Global School Health Survey [31]. The responses in the questionnaire were recorded by the number of days in the last 30 days for smoking cigarettes and smokeless tobacco and the number of times the participant consumed marijuana in the last 30 days. Due to lower proportion of adolescents reporting their usage in the last 30 days (6.1% smoking, 0.6% smokeless tobacco, and 9.8% marijuana), the final response of the participants for analysis was recorded as ‘no’ if the chosen option was 0 times/days, and was recorded as ‘yes’ for the rest of the options of use for all 3 substances. The Chronbach’s alpha for reliability of the coping strategy questionnaire was 0.70. Furthermore, the coping strategies, such as food-seeking strategies and the number of substances used are presented, stratified by drop-out of school status.

The individual (age, sex, education level, school enrolment and adolescent work status, COVID-19 infection history and coping strategy variables) and socio-environmental factors (number of household earning members, number of younger siblings, father’s work status, household status, number of household assets categories, food aid received in pandemic, COVID-19 infection history in family, household head sex, household head education and number of household members) of the participants were also included in this study.

### Statistical analysis

Data was analyzed using SPSS version 25. All individual and socio-environmental variables were categorized and descriptive statistics (frequency, percentage, mean and standard deviation) are presented. The four categories of FI variables (no, mild, moderate and severe) were consolidated to make 3 variables for further analyses: no/mild, moderate and severe FI. Chi-square test was used to analyze association between dependent and independent variables and significant variables (p < 0.05) were identified for logistic regression. Ordinal logistic regression was performed to identify the factors associated with the ordinal response of adolescent FI. Results are shown as adjusted odds ratio (AOR) and associated 95% CI with p < 0.05 considered to be significant. Furthermore, to measure the association between adolescent FI and coping strategies stratified by drop out of school status, Chi-square test was performed separately by drop out of school status (p < 0.05).

## Results

**Fig. 1:**
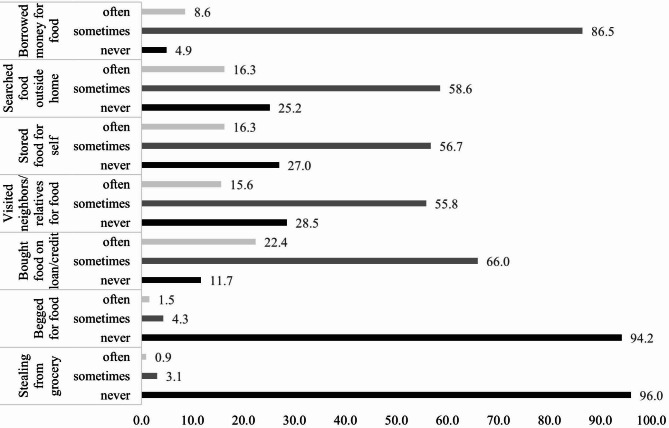
Food seeking coping strategies among adolescents

Of a total of 326 adolescents, while 2.2% was food secure, 23.6% reported mild, 46.6% moderate and 29.8% severe FI. Majority of participants were females (62.3%), early adolescence (12–14 years, 51.5%) and had some secondary education (50.6%) (Table 1). Regarding the types and frequencies of the various food seeking coping strategies (Fig. 1), most common coping strategies adopted by adolescents were borrowing money for food (95.1%) and buying food on loan and credit (88.4%), whereas begging (5.8%) and stealing (4%) were the least adopted coping strategies.

**Table 1:** Descriptive statistics for individual factors and their association with food insecurity levels

INDIVIDUAL FACTORS		All (n = 326)	No/mild (n = 77)	Moderate (n = 152)	Severe (n = 97)	P-value
		n (%)				
***Demographic factors***						
Age of adolescents (Mean ± SD: 14.5 ± 1.8)	Early adolescence (12–14 years)	168 (51.5)	35 (45.5)	72 (47.4)	61 (62.9)	**0.027**
	Late adolescence (15–18 years)	158 (48.5)	42 (54.5)	80 (52.6)	36 (37.1)	
Adolescent sex	Female	203 (62.3)	39 (50.6)	103 (67.8)	61 (62.9)	**0.041**
	Male	123 (37.7)	38 (49.4)	49 (32.2)	36 (37.1)	
Education level	None/some primary(1–5)	161 (49.4)	39 (50.6)	61 (40.1)	61 (62.9)	**0.002**
	Some secondary (6–12)	165 (50.6)	38 (49.4)	91 (59.9)	36 (37.1)	
School enrollment	Enrolled	286 (87.7)	63 (81.8)	139 (91.4)	84 (86.6)	0.102
	Not enrolled	40 (12.3)	14 (18.2)	13 (8.6)	13 (13.4)	
Adolescent work status	Not working	290 (92.6)	64 (81.8)	144 (94.7)	94 (96.9)	**0.001**
	Working	23 (7.4)	14 (18.2)	8 (5.3)	3 (3.1)	
COVID19 infection	No	303 (92.6)	72 (97.4)	145 (95.4)	86 (88.7)	0.126
history	Yes	23 (7.1)	5 (6.5)	7 (4.6)	11 (11.3)	
***Coping strategy factors***						
Stopped school due to lack of food/money	No	260 (79.8)	67 (87.0)	132 (86.8)	61 (62.9)	**< 0.001**
	Yes	66 (20.2)	10 (13.0)	20 (13.2)	36 (37.1)	
Food seeking	1st tertile (0–4)	108 (33.1)	35 (45.5)	60 (39.5)	13 (13.4)	**< 0.001**
strategies	2nd tertile (5–6)	138 (42.3)	24 (31.2)	67 (44.1)	47 (48.5)	
	3rd tertile (6–14)	80 (24.6)	18 (23.3)	25 (16.4)	37 (38.1)	
Smoking cigarettes last month	Yes	20 (6.1)	5 (6.5)	6 (3.9)	9 (9.3)	0.229
	No	306 (93.9)	72 (93.5)	146 (96.1)	88 (90.7)	
Marijuana last month	Yes	32 (9.8)	10 (13.0)	13 (8.6)	9 (9.3)	0.554
	No	294 (90.2)	67 (87.0)	139 (91.4)	88 (90.7)	
No.of substance	Two or more	7 (2.1)	0 (0.0)	2 (1.3)	5 (5.1)	**0.033**
used last month	At least one	41 (12.6)	15 (19.5)	17 (11.2)	9 (9.3)	
(Mean ± SD: 0.16 ± 0.409)	None	278 (85.3)	62 (80.5)	133 (87.5)	83 (85.6)	

Abbreviations: SD – Standard deviation

Bivariate analysis showed that individual factors (Table 1) (age, adolescent sex, education level, adolescent work status, and adolescents’ coping strategies, such as food seeking, drop out of school and number of substances used) and socio-environmental factors (Table 2) (number of households earning members, father’s working status, household status, number of household assets, food aid received in pandemic, COVID-19 infection of family members, household head sex and education, and number of household members) were associated with FI (p < 0.05).

**Table 2:** Descriptive statistics for socio-environmental factors and their association with 3 levels (no/mild, moderate, severe) of food insecurity

SOCIO-ENVIRONMENTAL FACTORS		All (n = 326)	No/mild (n = 77)	Moderate (n = 152)	Severe (n = 97)	P-value
		n (%)				
No. of earning members (Mean ± SD: 1.3 ± 0.457)	1 earning member	230 (70.6)	44 (57.1)	111 (73)	75 (77.3)	**0.010**
	> 1 earning member	96 (29.4)	33 (42.9)	41 (27.0)	22 (22.7)	
No. of younger siblings	None	138 (42.3)	33 (47.0)	63 (41.4)	42 (43.3)	0.957
(Mean ± SD: 0.8 ± 1.0)	1 sibling	130 (39.9)	29 (34.9)	61 (40.1)	40 (41.2)	
	> 1 sibling	58 (17.8)	15 (18.1)	28 (18.4)	15 (15.5)	
Father work status	Working	295 (90.5)	75 (97.4)	138 (90.8)	82 (84.5)	**0.016**
	Not working	31 (9.5)	2 (2.6)	14 (9.2)	15 (15.5)	
Household status	Not self-owned/rented	177 (54.3)	25 (32.5)	88 (57.9)	64 (66.0)	**< 0.001**
	Rented	84 (25.8)	22 (28.6)	34 (22.4)	28 (28.9)	
	Self-owned	65 (19.9)	30 (63.8)	30 (19.7)	5 (5.2)	
No. of household assets	No assets	230 (70.6)	31 (40.3)	113 (74.3)	86 (88.7)	**< 0.001**
(savings, jewelry, electronics	1 asset category	67 (20.6)	21 (27.3)	36 (23.7)	10 (10.3)	
/home appliances, vehicles)	>=2 asset categories	29 (8.8)	25 (32.4)	3 (2.0)	1 (1.0)	
Food aid received in pandemic	Some food aid	276 (84.7)	56 (72.7)	132 (86.8)	88 (90.7)	**0.003**
	No food aid	50 (15.3)	21 (27.3)	20 (13.2)	9 (9.3)	
COVID 19 infection	No	301 (92.3)	75 (97.4)	145 (95.4)	81 (83.5)	**< 0.001**
history in family	Yes	25 (7.7)	2 (2.6)	7 (4.6)	16 (16.5)	
Household head sex	Female	22 (6.7)	2 (2.6)	8 (5.3)	12 (12.4)	**0.023**
	Male	304 (93.3)	75 (97.4)	144 (94.7)	85 (87.6)	
Household head	No education	242 (74.2)	56 (72.7)	117 (77.0)	69 (71.1)	**0.031**
education	Some primary	60 (18.4)	10 (13.0)	26 (17.1)	24 (24.7)	
	Some secondary	24 (7.4)	11 (14.3)	9 (5.9)	4 (4.1)	
No. of household	< 5	103 (31.6)	33 (42.9)	39 (25.7)	31 (32.0)	**0.03**
members (Mean ± SD: 5.26 ± 1.525)	>=5	223 (68.4)	44 (57.1)	113 (74.3)	66 (68.0)	

Abbreviations: SD – Standard deviation

As seen in Table 3, significant predictors (p < 0.05) for adolescent FI include individual factors such early adolescents (12–14 years) and adolescents with lower education level (no education/some primary) were 1.7 times (95% CI [1.1, 2.5]) and 1.5 times (95% CI [1.1, 2.3]) more likely to be food insecure compared to older adolescents (15–18 years) and those with some secondary education respectively. Employed adolescents in our study were found to be 5.4 times (95% CI [2.3, 12.7]) less likely to suffer from a higher degree of FI. Socio-environmental factors such as female household head (AOR 4.7, 95% CI [2.0, 11.4]) and lower education of household head (AOR 4.2, 95% CI [1.7, 10.6]) were strong predictors of adolescent FI. Other significant socio-environmental factors that play are role in determining the adolescent FI in a household includes ≥ 5 family members (AOR 1.7, 95% CI [1.1, 2.9]), only 1 earning family member(AOR 2.6, 95% CI [1.6, 4.2]), unemployed father (AOR 3.0, 95% CI [1.5, 6.2]) and no household assets (AOR 39.7, 95% CI [13.1, 120.5]). Adolescents who received some food aid during the COVID-19 pandemic were 2.6 times (95% CI [1.4, 4.6]) more food insecure that those who did not receive food aid during the pandemic. Positive COVID-19 infection in family (AOR 4.7, 95% CI [2.0, 10.9]) was another significant predictor to adolescent FI. As seen, adolescents who suffered a higher level of FI, have higher likelihood of adopting more coping strategies. They were 2.6 times (95% CI [1.6, 4.2]) more likely to be on the second tertile and 3.4 times (95% CI [1.9, 5.9]) more likely to be on the third tertile for use of food seeking related coping strategies. They are also 6.2 times (95% CI [1.2, 31.7]) more likely to use two or more substances and 3.3 times (95% CI [1.9, 5.7]) more likely to stop school.

**Table 3:** Summary statistics using ordinal logistic regression of adjusted odds ratio (AOR) and 95% Confidence interval (CI) for individual, socio-environmental and coping strategy variables in association with food insecurity

		Food insecurity (No/mild = 0, Moderate = 1, Severe = 2)	
		AOR (95% CI)	p-Value
**Individual factors**			
Age of adolescents	Early adolescence (12–14 years)	1.658 (1.101–2.498)	**0.016**
	Late adolescence (15–18 years)	Reference	
Adolescent sex	Male	1.416 (0.924–2.169)	0.110
	Female	Reference	
Adolescent education level	No education-some primary(1–5)	1.525 (1.011–2.302)	**0.044**
	Some secondary(6–12)	Reference	
Adolescent work status	Not working	5.365 (2.265–12.711)	**< 0.001**
	Working	Reference	
**Socio-environmental factors**			
No. of household members	>=5	1.734 (1.043–2.881)	**0.034**
	< 5	Reference	
Household head sex	Female	4.728 (1.958–11.413)	**0.001**
	Male	Reference	
Household head education	No education Some primary	2.590 (1.146–5.856) 4.232 (1.682–10.646)	**0.022** **0.002**
	Some secondary	Reference	
No. of household earning members	1 earning member	2.593 (1.594–4.216)	**< 0.001**
	> 1 earning member	Reference	
Father work status	Not working	2.988 (1.450–6.156)	**0.003**
	Working	Reference	
Household status	Neither self-owned nor rented	4.973 (2.843–8.697)	**< 0.001**
	Rented	3.402 (1.810–6.396)	**< 0.001**
	Self-owned	Reference	
No. of household assets	1 asset	12.799 (4.035–40.596)	**0.001**
(savings, jewelry, electronics	no assets	39.653 (13.047–120.517)	**0.001**
/home appliances, vehicles)	>=2 asset	Reference	
Food aid received in pandemic	Some food aid	2.561 (1.425–4.604)	**0.002**
	No food aid	Reference	
History of COVID 19 infection in family	Yes	4.697 (2.023–10.903)	**< 0.001**
	No	Reference	
**Coping strategies**			
Food seeking strategy	3rd tertile (6–14)	3.353 (1.897–5.926)	**0.001**
	2nd tertile (5–6)	2.582 (1.602–4.159)	**< 0.001**
	1st tertile (0–4)	Reference	
Stopped school due to lack of food/money	Yes	3.300 (1.921–5.669)	**< 0.001**
	No	Reference	
No. of substance used last month	Two or more	6.186 (1.209–31.642)	**0.029**
	At least one	0.555 (0.297–1.039)	0.066
	None	Reference	

Abbreviations: SD – Standard Deviation, AOR – Adjusted Odds Ratio, CI – Confidence Interval

Furthermore, when adolescent coping strategies are stratified by status, drop out of school were assessed (Table 4), a positive association between FI levels and food seeking strategies were observed among those school-going (p < 0.001), while there was no association among those dropping out of school (p = 0.100). In terms of number of substances used the previous month, there were no associations, regardless of drop-out of school status (p > 0.05). Although, severe FI had a higher percentage of marijuana use the previous month, compared to their counterparts.

**Table 4:** Adolescent coping strategies by status of drop-out of school

	Drop out of school (no)			p-value	Drop out of school (yes)			p-value
	No/mild n (%)	Moderate n (%)	Severe n (%)		No/mild n (%)	Moderate n (%)	Severe n (%)	
**Food seeking**				**< 0.001**				0.100
1st tertile (0–4)	31 (46.3)	54 (41.0)	8 (13.1)		4 (40.0)	6 (30.0)	5 (13.9)	
2nd tertile (5–6)	22 (32.8)	56 (42.4)	31 (50.8)		2 (20.0)	11 (55.0)	16 (44.4)	
3rd tertile (6–14)	14 (20.9)	22 (16.6)	22 (36.1)		4 (40.0)	3 (15.0)	15 (41.7)	
**Smoking cigarettes last month**								
Never	64 (95.5)	128 (97.0)	56 (91.8)	0.282	8 (80.0)	18 (90.0)	32 (89.0)	0.704
Yes	3 (4.5)	4 (3.0)	5 (8.2)		2 (20.0)	2 (10.0)	4 (11.0)	
**Marijuana use**								
**last month**								
Never	57 (85.0)	122 (92.4)	58 (95.0)	0.105	10 (100.0)	17 (85.0)	30 (83.3)	0.388
Yes	10 (15.0)	10 (7.6)	3 (5.0)		0 (0.0)	3 (15.0)	6 (16.7)	
**No of substance**								
**used last month**								
None	54 (80.6)	118 (89.3)	53 (86.9)	0.295	8 (80.0)	16 (80.0)	30 (83.3)	0.315
At least one	13 (19.4)	14 (10.7)	8 (13.1)		2 (20.0)	2 (10.0)	1 (2.8)	
Two or more	0 (0.0)	0 (0.0)	0 (0.0)		0 (0.0)	2 (10.0)	5 (13.9)	

## Discussion

To our knowledge, this study is the first to report adolescent-reported FI and the association with individual and socio-environmental factors and coping strategies in urban slums in Bangladesh during the times of the pandemic. Two-third reported moderate or severe FI and they are more inclined to a wider selection of coping strategies, such as food seeking, substance use and stopping school due to lack of food/money. This study showed that adolescents from underprivileged households are at very high risk of FI and resort to coping strategies.

The prevalence of adolescent FI in our study was found to be much higher compared to those from previous studies in other LMICs. A recent study on FI among adolescent students from 95 countries using data from the Global School-based School Health Survey (GSHS) showed that 25.5% aged 11–14 years compared with 30% aged 15–18 years reported FI [20]. Another from low income countries such as Pakistan [10] and Southwest Ethiopia [11] was reported to be around 50%. The high prevalence noted in our study may be due to the study timing, since our study was conducted during the COVID-19 pandemic. COVID-19 has exacerbated malnutrition and FI at a global scale [32]. Adolescents living in LMICs were therefore at a higher risk of FI during the COVID-19 pandemic, attributed to loss of income of the low-income families during the global and nationwide recession in Bangladesh during the pandemic [33]. Insufficient efforts to deal with this situation, lack of adequate understanding and screening for adolescent FI, have only increased the burden of the situation in the pandemic [32]. In addition, the recent humanitarian crisis events and inflation has led to the International Monetary Fund (IMF) categorizing Bangladesh as one of the hunger hotspots [34]. To lessen the impact of the existing adolescent FI problem, policymakers should prioritize battling current inflation and safeguarding the most disadvantaged which includes adolescents residing in urban slums.

Adolescent FI may be an indicator for a wide set of individual, social, and household challenges that contribute to adolescent health and well-being. Since the legal age of employment in Bangladesh is 14 years [35], older adolescents particularly from socially disadvantaged households can support themselves financially and also provide for their families. Our study supported this by showing that older adolescents and those working are less food insecure. However, When children are compelled to leave school and engage in labor due to economic pressure, they are deprived of the opportunity to acquire skills and capabilities essential to realize their full potential, securing stable and well-paying jobs and disrupt a cycle of disadvantage and poverty [35]. Both dropping out of school and having a lower level of education can therefore increase the likelihood and eventually worsen the cycle of adolescent FI. This is also further supported by strong associations of adolescent FI with socio-economic factors, such as father’s unemployment, low household assets possession, lower number of households’ earning members, female led households, and household heads with lower education as shown in previous researches [8, 9, 11]. Studies in developing countries such as Bangladesh have shown that, female-led households are often more vulnerable to experiencing FI [8, 36]. Less employment opportunities were available to households headed by women, and their pay rates were lower than those of male led households. In many instances, females also bear the burden through assigned social norms regarding the nature of work they can or cannot do as well as domestic and childcare responsibilities [8]. Social reform policies should be implemented to ensure more women are included in workforce and close the gender inequality gap. This study found no gender link to FI. However, Sheikh et al. in his study found adolescent boys more prone to it than girls [10]. A study in Ethiopia suggested higher FI among adolescent girls [11]. These conflicting results highlight the need to understand what is perceived as FI. Findings from a study conducted in Bangladesh showed that interaction between gender norms and structural, social, and economic factors often predispose female adolescents to poor nutritional consumption [37]. According to the study, young female respondents found satisfaction in offering food first to the males in their households, accepting their less significant status and the financial restraints of their families, which caused them to habitually consume less food than their physical needs [37]. This could have likely resulted in under reporting of FI by adolescent females. Therefore understanding of FI can vary depending on the environment and context of the study. Furthermore, the current study showed a higher family size was substantially related to adolescent FI. This may be mostly because in poor families from slums, food expenditure constitutes a large percentage of total household expenditures [3]. Therefore, having bigger families could lead to adolescents compromising their dietary needs despite higher nutritional demands [14]. The positive association with COVID-19 infection in family can be explained by the fact that poorer communities are often more susceptible to severe disease once it is contracted and this could have resulted in greater income loss due to work disruptions for the adolescent households, thereby worsening their state of FI [26]. Our study also showed that although about 85% of slum-dwelling adolescents have ever received food aids in the COVID-19 pandemic (90% in severe FI, 86.8% in moderate FI and 72.7% in no/mild FI adolescents, prevalence of the overall adolescent FI in our study was still incredibly high. Bangladesh’s social security policies rarely addressed specific adolescent needs and for the enormous population living in poverty, the government has been unable to provide enough aid and social assistance to the vulnerable during the pandemic [33]. This shows that in order to be effective, policies need to be more targeted and designed to address specific needs of vulnerable population. Different from our study showing a positive association of FI with socioeconomic factors, a study done on adolescents in rural Pakistan showed no significant associations [10], and suggested that FI requires a multilevel investigation of other variables such as social support, food prices, unforeseen events like medical or other costs, etc. rather than blaming it all on poverty [10].

While some studies explored coping strategies in adolescents, they lack inference based on quantitative analysis [19]. In our opinion, our study is the first to have quantitatively analyzed the association between adolescent FI and three types of coping strategies which are food seeking coping, substance use coping and dropping out of school. Our study revealed that severely food insecure adolescents are more likely than their counterparts to utilize a larger variety of coping methods to deal with FI, such as a higher number of food-seeking strategies as well as number of substances used and dropping out of school. Adolescents dropping out of school due to lack of foods were around 20% and the drop-out rate increases as the severity of FI increases. This may indicate that drop out of school status can increase the likelihood and in due course, worsen the state of FI and subsequent coping strategies. Interestingly, food seeking strategies used were also different by status of stopping school. While a significant association between food seeking strategies and FI remained significant among school-going adolescents, there was no association among adolescents dropping out of school. It may indicate that different from adolescents being employed, school-going adolescents are still struggling to cope with FI. The government’s safety net programs in Bangladesh to reduce FI, largely include food transfer programs which are all food-focused [38]. A study in Bangladesh showed that cash transfer programs have been more beneficial in increasing caloric intake among school age children compared to food transfer programs [38]. Another study showed that school feeding programs to improve FI, will not only improve health in adolescents but also increase the number of school days attended [39]. Therefore, it is highly recommended that policy makers modify their current safety net programs and incorporate more diversified policies and strategies that are designed to address the various issues and factors associated with FI.

In lights of substance use, although the study from the GSHS from 95 countries revealed that FI is associated with a higher odds of substance use, such as smoking, drinking and drugs [20], our study did not show a significant association with neither smoking cigarettes nor marijuana, but has shown a positive association between adolescent FI and the number of substances used last month. This shows that FI may result in greater experimentation with tobacco and marijuana use. Adolescents often use substances as source of relief from any mental stress and anxiety arising from difficult situations in their lives with unawareness of the consequences [2, 40] and as a sense of belonging with their peers [20]. Our finding is important, as it is crucial to identifying adolescents at earliest stage of FI, to prevent worsening of their FI state which could lead to detrimental substance abuse among youths from disadvantaged populations who may be at a higher risk of drug abuse and subsequent poor health [20]. While there was no association of smoking cigarettes and marijuana use with FI, regardless of drop-out of school status, percentages of marijuana use seem higher in severe food insecure group among adolescents stopping school. Given the results of this study, interventions to reduce FI may need to be tailored to coping strategies that differ by adolescent age, sex and school-going status. Since understanding the use of coping strategies enable us to understand the extent to which individuals can go in improving their situation, it can help implement policies that are precisely designed to tackle and improve FI situation among adolescents. Yet, our results need to be further examined because these mechanisms can be situation or context specific.

Our study’s advantages include data on adolescents from urban slum communities with self-reported estimates of FI and various levels of determinants since the literature on adolescent FI is relatively inadequate in LMICs, especially in urban slum areas. Nonetheless, some limitations of the study should be acknowledged. Due to the nature of a cross-sectional study design, a causal relationship cannot be assumed and recall bias may be introduced. Additionally, this survey was also collected during the monsoon season (beginning May) in Bangladesh when household FI is more likely to be high in comparison to the dry season [41]. This study only included adolescents and their households who could converse with researchers in the standard spoken Bengali, this could have underestimated the FI. Since slum households were limited in their space and privacy, constraints to how much privacy could be maintained between participants and their caregivers could have introduced response bias to some sensitive questions (e.g. smoking, begging etc.). In addition, although HFIAS questionnaire has been used in various studies in Bangladesh [7, 27] and validated for use in other LMICs [28, 29], it is yet to be linguistically and cross-culturally validated for use in Bengali language.

## Conclusion

Adolescent-reported FI in urban slums of Bangladesh was found to be remarkably high. The participants shared a wide range of coping strategies to neutralize risks of FI such as food seeking strategies, substance use and pause in schooling. Adolescent FI is a critical aspect of SDG 2, as it highlights the need to address hunger, malnutrition, and inadequate access to nutritious food of adolescents. As seen with this study, adolescent FI is influenced by a combination of individual, social and economic factors. In order to achieve SDG 2, it is important to recognize these factors and address them through comprehensive and multi-dimensional approach which involves concerted efforts from numerous shareholders such as the government, private institutions and non-governmental organizations. Therefore, prioritizing adolescent FI in developing countries such as Bangladesh can have far reaching implications that touch on education, economic development and overall health and wellbeing and thus contribute to more productive lives for young people in Bangladesh as well as other developing nations globally.

## Data Availability

All data generated or analyzed during this study are included in this article.

## Abbreviations

AOR: Adjusted Odds Ratio
CI: Confidence Interval
COVID-19: Coronavirus 19
FI: Food Insecurity
GSHS: Global School-based School Health Survey
HFIAS: Household Food Insecurity Access Scale
IMF: International Monetary Fund
LMIC: Low to Middle Income Country
SD: Standard deviation
SDG: Sustainable Development Goal
